# Isokinetic eccentric vs. concentric training for functional ankle instability: a randomized controlled trial

**DOI:** 10.1186/s13018-025-06194-0

**Published:** 2025-08-20

**Authors:** Dunbing Huang, Wei Song, Yang Liu, Xiaohua Ke, Zhenhua Wu

**Affiliations:** 1 Encephalopathy and Rehabilitation Center, The Second Affiliated Hospital of Zhejiang Chinese Medical University, No. 318 Chaowang Road, Hangzhou, Zhejiang 310005 China; 2 Department of Rehabilitation Medicine, Shanghai Fourth People’s Hospital, School of Medicine, Tongji University, 1279 Sanmen Road, Shanghai, 200434 China; 3 Department of Rehabilitation Medicine, Zhejiang Hospital, No. 12 Lingyin, Zhejiang 310013 Hangzhou, China

**Keywords:** Isokinetic eccentric training, Isokinetic muscle strength, Functional ankle instability, Postural control, Dorsiflexion, Eversion

## Abstract

**Objective:**

To investigate the effects of a 12-week isokinetic eccentric training program on postural control and ankle muscle strength in individuals with functional ankle instability (FAI).

**Methods:**

In this randomized controlled trial, 42 participants with unilateral FAI were randomly assigned to either an experimental group (*n* = 21), receiving isokinetic eccentric training, or a control group (*n* = 21), receiving isokinetic concentric training. Both groups trained three times per week for 12 weeks. One participant from each group dropped out during the intervention, resulting in 20 participants per group included in the final analysis. Primary outcomes included static and dynamic postural control assessed using the Pro-Kin 254P platform. Secondary outcomes were ankle dorsiflexion and eversion strength, as well as dorsiflexion/plantarflexion (D/P) and eversion/inversion (E/I) torque ratios measured with a Biodex System 4 Pro^®^ dynamometer at 60°/s and 180°/s. Between-group comparisons and effect sizes (Cohen’s d) with 95% confidence intervals (CI) were reported.

**Results:**

After 12 weeks, the experimental group demonstrated significantly greater improvements in postural control and ankle strength outcomes compared to the control group (*p* < 0.01). For dynamic postural control, the total offset index decreased by 23.8% in the eccentric group, showed a significant improvement with a very large effect size (Cohen’s d = 2.06, 95% CI [1.29, 2.82], *p* < 0.001). Static postural control, measured by sway area, was reduced by 17.3% (Cohen’s d = 0.81, 95% CI [0.16, 1.45], *p* = 0.015), while sway length did not reach statistical significance (Cohen’s d = 0.55, 95% CI [–0.08, 1.18], *p* = 0.090). Regarding ankle strength, dorsiflexion relative peak torque (RPT) at 60°/s increased by 30.7% in the eccentric group (Cohen’s d = − 0.79, 95% CI [–1.44, − 0.15], *p* = 0.016), while eversion RPT at 180°/s improved by 75.7% (Cohen’s d = − 1.13, 95% CI [–1.79, − 0.46], *p* = 0.001). The D/P torque ratio at 180°/s exhibited a very large between-group difference (Cohen’s d = − 2.18, 95% CI [–2.96, − 1.39], *p* < 0.001), and the E/I torque ratio at 60°/s demonstrated an extremely large effect size (Cohen’s d = − 4.50, 95% CI [–5.67, − 3.34], *p* < 0.001).

**Conclusion:**

Isokinetic eccentric training significantly enhances postural stability and ankle muscle strength in patients with FAI. These improvements in torque symmetry and balance support the inclusion of eccentric training as an effective rehabilitation strategy to reduce reinjury risk and restore neuromuscular function.

## Introduction

Ankle sprains are one of the most prevalent musculoskeletal injuries, accounting for approximately 20% of all sports-related injuries [1–3]. These injuries frequently occur during dynamic activities such as jumping, cutting, and landing, particularly in sports like basketball, soccer, and volleyball [4, 5]. While acute ankle sprains often resolve with conservative management, up to 40% of patients develop chronic ankle instability (CAI), characterized by recurrent giving-way episodes, pain, and functional limitations [6–8]. CAI is further classified into functional ankle instability (FAI), which involves sensorimotor deficits without structural ligamentous laxity, and mechanical ankle instability (MAI), which results from anatomical damage to the stabilizing ligaments [9]. FAI has attracted growing interest due to its complex neuromuscular origins and subtle clinical signs. Unlike MAI, which can often be identified via imaging and physical tests, FAI is harder to diagnose, as it primarily stems from impaired neuromuscular function rather than structural damage. This condition leads to long-term deficits in neuromuscular control, postural stability, and muscle activation, thereby increasing the risk of reinjury and secondary osteoarthritis [10–12].

A key factor contributing to FAI is the disruption of agonist-antagonist muscle balance around the ankle joint, particularly between the dorsiflexors and plantarflexors, and between the evertors and invertors [13]. This imbalance compromises dynamic joint stability by altering force production and motor control. Patients with FAI often show significant weakness in the dorsiflexors and evertors, resulting in abnormal dorsiflexion/plantarflexion (D/P) and eversion/inversion (E/I) torque ratios. These altered ratios negatively impact joint stabilization mechanisms, especially during high-demand tasks such as cutting, jumping, or sudden directional changes, which are common in athletic activities. As a result, patients demonstrate increased postural sway and a diminished capacity to recover from external perturbations, both of which contribute to a heightened risk of recurrent ankle injuries [14]. Moreover, FAI is consistently linked to proprioceptive deficits and delayed activation of the peroneal muscles, which are vital for rapid lateral stabilization during inversion movements [15, 16]. These neuromuscular deficits not only compromise static and dynamic balance but may also interfere with central sensorimotor integration. Given the critical role of ankle musculature in maintaining postural control, interventions that restore muscle strength, correct imbalances, and improve neuromuscular coordination are essential for reducing reinjury risk and optimizing outcomes.

Traditional FAI rehabilitation focuses primarily on balance and proprioceptive training to enhance postural control and prevent recurrence [17–19]. While effective for improving coordination and joint position sense, these methods often fail to address the associated muscle strength deficits and imbalances. Concentric strength training, a common component of traditional rehabilitation, can induce muscle hypertrophy and increase force output. However, it may be insufficient in enhancing neuromuscular control or developing eccentric strength, which is essential for resisting and decelerating ankle inversion forces during dynamic athletic movements [20]. Eccentric training has gained attention for its superior ability to promote neural adaptations, including enhanced motor unit recruitment, greater corticospinal excitability, and increased musculotendinous stiffness, factors essential for dynamic joint stability [21]. Among eccentric methods, isokinetic eccentric training offers distinct advantages, such as constant angular velocity and precise loading throughout the range of motion, minimizing compensatory movement patterns [22]. Recent evidence supports its effectiveness in restoring muscle balance, improving D/P and E/I strength ratios, and enhancing functional outcomes in patients with musculoskeletal impairments. However, despite its theoretical and practical benefits, no randomized controlled trials (RCTs) to date have systematically investigated the effects of isokinetic eccentric training on both postural control and ankle strength ratios in individuals with FAI. This lack of high-quality evidence highlights a critical gap in current rehabilitation practices and underscores the need for rigorous clinical trials.

Therefore, this study aimed to investigate whether a 12-week isokinetic eccentric training program improves static and dynamic postural control and ankle strength ratios in individuals with functional ankle instability. It was hypothesized that eccentric training would lead to significant improvements in both postural control and ankle strength ratios, specifically the D/P and E/I torque ratios at 60°/s and 180°/s, ultimately enhancing functional stability. By directly comparing eccentric and concentric training modalities, this study seeks to inform evidence-based rehabilitation strategies and contribute to a more targeted and mechanism-driven approach to FAI management.

## Methods

### Study design and setting

This investigation was designed as a parallel-group, randomized controlled trial conducted at Zhejiang Hospital. The study protocol was approved by the Zhejiang Hospital Ethics Review Committee (No. 2023–34 K) and was registered with the Chinese Clinical Trial Registry (ChiCTR2500097380). All procedures adhered to the principles outlined in the Declaration of Helsinki. Written informed consent was obtained from all participants. Outcome assessors and data analysts were blinded to group allocation.

## Participants and sample size calculation

Participants were recruited from the outpatient department of Zhejiang Hospital between June 2023 and September 2024. A priori sample size calculation was performed using PASS software, based on data reported by Hanci et al. [23], where the mean ankle dorsiflexor strength at 300°/s was 18.5 ± 0.8 Nm in the eccentric training group and 17.3 ± 0.5 Nm in the control group. Using these values, a moderate effect size (Cohen’s d = 0.68) was estimated. To detect this effect with 92% statistical power and a two-tailed α level of 0.001, the minimum required sample size was calculated as 17 participants per group. To account for an anticipated 20% dropout rate, the final sample size was increased to 21 participants per group to maintain sufficient statistical power.

## Inclusion criteria

CAI, which includes both FAI and MAI subtypes, is currently diagnosed based on unified criteria without subtype distinction, as proposed by the International Ankle Consortium in 2014 [24]. In this study, participants were selected according to these CAI criteria but exhibited symptoms consistent with FAI. Participants who met the following inclusion criteria will be recruited: (1) aged ≥ 18 years old; (2) a history of at least one ankle sprain that caused discomfort, edema, and stiffness and prevented them from engaging in leisure or other activities or sports for at least three weeks; (3) recurrent ankle sprain (2 or more sprains in the same ankle) or feeling of instability during daily life activities in the previously injured ankle within 1 year prior to participation in the study; (4) Cumberland Ankle Instability Tool (CAIT) scores ≤ 24; (5) in the past year, there has been no engagement in any regular physical exercise (at least 20 min per session, at least twice a week) or any systematic isokinetic eccentric training.

## Exclusion criteria

Participants who met the following exclusion criteria were excluded: (1) previous fracture or surgery of the lower limb; (2) known instability of the trunk or other joints of the lower limb; (3) systemic neuromuscular disorders, abnormalities of the vestibular system or visual deficits; (4) mechanical ankle instability (positive test results on both the ankle anterior drawer test and talar tilt test); and (5) bilateral functional ankle instability (excluded via clinical examination including anterior drawer and talar tilt tests on both ankles).

## Withdrawal from the study

In any of the following cases, participants were allowed or asked to withdraw from the study: (1) Those unwilling to continue to participate in the trial at any stage of the trial for any reason. (2) Those who experience an adverse event (AE) that is not suitable for continued participation in the trial. (3) Participants who missed more than 25% of the training sessions (i.e., over 9 of 36) were withdrawn from the trial, consistent with prior protocols and existing literature on exercise adherence [17].

### Randomization and blinding

The participants were randomly allocated to either the experimental or control group using a randomization sequence generated by a researcher not involved in data collection or intervention. Allocation concealment was ensured through the use of sequentially numbered, opaque-sealed envelopes, which were opened only after recruitment and baseline data collection were completed. Although participant and therapist blinding were not feasible due to the intervention design, performance bias was minimized by conducting one-on-one sessions in a controlled environment. The same therapist supervised each participant using standardized protocols, and neutral instructions were provided to avoid differential encouragement. Furthermore, outcome assessors and data analysts remained blinded to group allocation throughout the study to ensure objective outcome evaluation.

## Interventions

All training sessions were conducted under the supervision of professionally trained rehabilitation therapists and lasted approximately 60 min, administered three times per week for 12 consecutive weeks. Participant adherence was monitored using attendance records and therapist checklists. The training followed standardized protocols developed by an interdisciplinary rehabilitation research team, based on current scientific literature [25]. The exercises were specifically designed to address muscle imbalances commonly observed in individuals with FAI, particularly targeting weaknesses in the dorsiflexor and evertor muscle groups. A summary of the training components for both the experimental and control groups, including warm-up, main training and cool-down procedures, is provided in Table 1.

**Table 1:** Detailed exercise protocols for the experimental and control groups

Stage	Experimental group	Control group
**Warm-up**	Static stretches for all ankle directions (30s x 3 reps), treadmill walk 5 min, 20 repetitions of bipedal jump-landings	Same
**Main training**		
Equipment	Biodex System 4 Pro^®^ isokinetic dynamometer	Same
Pattern	Isokinetic eccentric	Isokinetic concentric
Movements	Eversion/Inversion + Dorsiflexion/Plantarflexion	Same
Angular Velocities	60°/s → 120°/s → 180°/s → 120°/s → 60°/s	Same
Repetitions	10 reps per velocity × 5, 50 reps per set, 2 sets	Same
Intensity	Weeks 1–4: 70% MVIC Weeks 5–8: 80% MVIC Weeks 9–12: 90% MVIC	Same
Rest Intervals	1 min between speeds 3 min between sets	Same
**Cool-down**	Static stretches for all ankle directions, 30s x 3 reps	Same

reps, repetitions; s, second; min, minute; MVIC, maximal voluntary isometric contraction

All training sessions were conducted on an individual basis rather than in groups. Each participant was supervised by the same therapist throughout the 12-week intervention to ensure consistency in instruction and minimize therapist-related bias across both the eccentric and concentric training groups.

## Experimental group

**Fig. 1:**
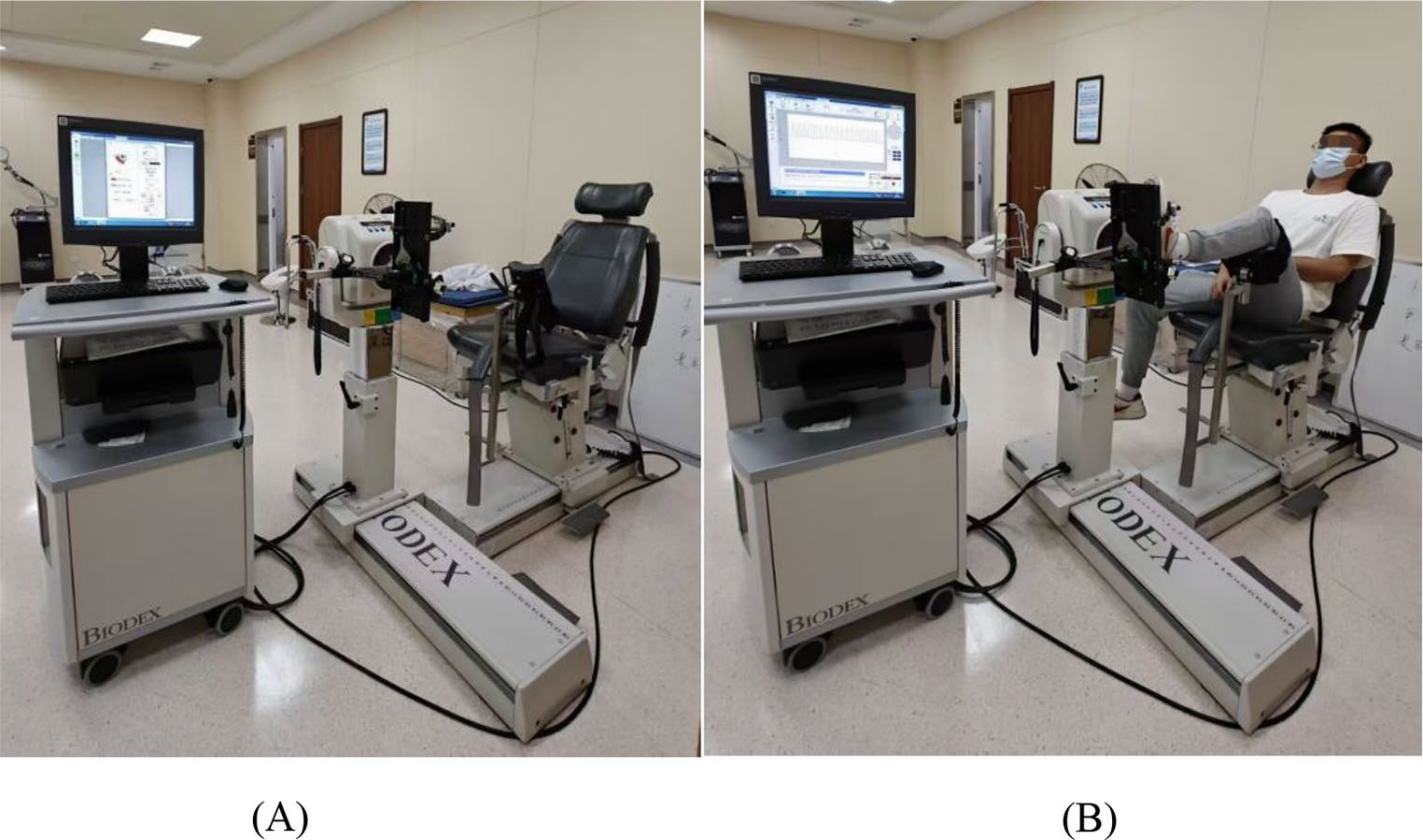
(**A**) Biodex System 4 Pro^®^ isokinetic dynamometer. This isokinetic dynamometer provides controlled angular velocity and precisely measures ankle joint muscle performance, including peak torque and strength ratios. (**B**) Isokinetic training and test of ankle joint muscle strength. The participant was seated with the knee flexed at 45° and the ankle positioned at neutral (0°), ensuring proper alignment of the lower limb with the dynamometer’s axis of rotation for training and testing

Participants in the experimental group received isokinetic eccentric training targeting the ankle dorsiflexor and evertor muscles, administered using a Biodex System 4 Pro^®^ isokinetic dynamometer (Biodex Medical Systems Inc., USA) (Fig. 1A). To minimize the risk of muscle damage and delayed onset muscle soreness associated with eccentric training, the maximal voluntary isometric contraction (MVIC) of the dorsiflexor and evertor muscles of the affected ankle was assessed prior to the first session [22]. Training intensity was then progressively adjusted based on this baseline, with participants exercising at 70% MVIC during weeks 1–4, 80% MVIC during weeks 5–8, and 90% MVIC during weeks 9–12.

Each session began with a standardized warm-up including static stretching of the ankle dorsiflexor, plantarflexor, invertor, and evertor muscles (30 s per stretch, three repetitions per direction), followed by five minutes of treadmill walking and 20 repetitions of bipedal jump-landings. A standardized rest interval of 5 min was provided between the warm-up and the main training phase to ensure adequate recovery and minimize fatigue effects. The main training protocol involved eccentric contractions in eversion/inversion and dorsiflexion/plantarflexion patterns using a progressive angular velocity sequence (60°/s, 120°/s, 180°/s, 120°/s, 60°/s), with 10 repetitions per speed, totaling 50 repetitions per set and two sets per session. Standardized rest intervals were 1 min between velocities and 3 min between sets (Fig. 1B). Cool-down included seated passive stretches for all ankle muscle groups using the same standardized parameters as in the warm-up. The intervention was applied unilaterally to the pathological (affected) ankle only, reflecting the clinical presentation of unilateral FAI.

### Control group

The control group completed isokinetic concentric training under the same frequency, duration, warm-up, and cool-down conditions as the experimental group. A 5-minute rest was also provided after the warm-up to ensure recovery before training. Training was performed using the same Biodex System 4 Pro^®^ isokinetic dynamometer, targeting the ankle dorsiflexor and evertor muscles through identical movement patterns (eversion/inversion and dorsiflexion/plantarflexion) and the same angular velocity progression (60°/s, 120°/s, 180°/s, 120°/s, 60°/s) (Fig. 1B). Intensity was progressively adjusted based on each participants’ pre-assessed MVIC: 70% in weeks 1–4, 80% in weeks 5–8, and 90% in weeks 9–12. The only distinction from the experimental group was the use of concentric instead of eccentric muscle contractions. The warm-up and cool-down protocols, including the stretching routine, matched those used in the experimental group to ensure consistency across groups. As with the experimental group, the isokinetic concentric training was performed unilaterally on the affected limb only.

### Outcome measurements

Two trained assessors, blinded to group allocation, conducted evaluations within three days before and after the intervention. Each assessor was consistently responsible for specific outcome domains (i.e., one for postural control, the other for isokinetic muscle strength) throughout the entire study period to reduce measurement variability and ensure consistency across time points. To minimize bias, both assessors received standardized training and conducted all outcome evaluations independently and in a blinded manner, strictly following uniform measurement protocols.

### Primary outcomes

**Fig. 2:**
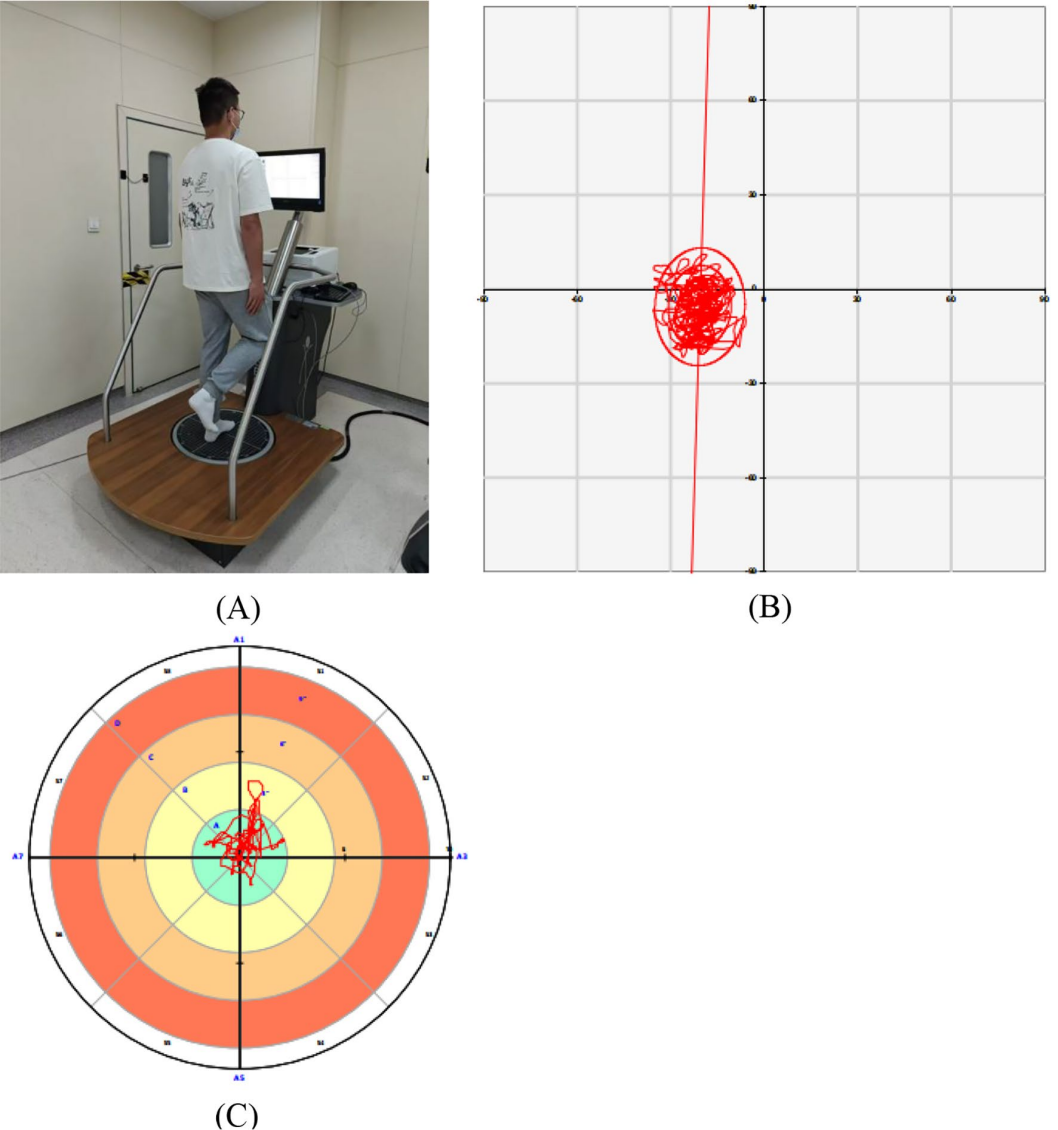
(**A**) Testing scene for postural control assessment using the Pro-Kin 254P platform. A participant performing single-leg stance testing during postural control evaluation. The assessment was conducted using the Pro-Kin 254P system under standardized laboratory conditions. The subject maintained balance barefoot while tracking instructions shown on the screen. (**B**) Static postural control results as measured on the Pro-Kin 254P platform. The red trace represents the center of pressure trajectory during quiet single-leg standing with eyes open. The data reflect sway behavior in a fixed base of support condition. A smaller and more centralized center of pressure trace indicates greater static postural stability. (**C**) Dynamic postural control results as measured on the Pro-Kin 254P platform. The red trace represents the participant’s center of pressure trajectory during dynamic balance testing. The concentric zones indicate distance from the target center, with the inner green area representing optimal balance control. A more centralized trace suggests better dynamic postural control and neuromuscular responsiveness

Postural control of the affected limb was assessed using the Pro-Kin 254P system (TecnoBody, Italy), a validated and reliable device for evaluating both static and dynamic balance in patients with chronic ankle instability [26]. The system uses force platform technology to detect real-time center of pressure (COP) displacements, which reflect postural stability [27]. The COP represents the weighted average of all ground reaction forces exerted through the foot and is considered a sensitive indicator of neuromuscular control. During testing, participants stood on the platform with their knees flexed at approximately 15°, eyes open, and arms relaxed at their sides while fixating on the visual target. Foot position coordinates were recorded to ensure test-retest consistency (Fig. 2A).

Static Stability Test: Participants stood on the affected leg atop the locked platform while focusing on a stationary target. Each trial lasted 30 s. The measured outcomes were total sway area (mm²) and total sway length (mm). Lower values indicate better static balance (Fig. 2B). These parameters have demonstrated strong reliability and are widely accepted in balance assessments [28]. 

#### Dynamic stability test

Conducted on the unlocked platform, this test required participants to maintain balance on the affected leg while following a moving visual target for 20 s. The key metric was the total offset index (°), which represents the total angular deviation of the COP from the platform center. Higher values indicate greater postural instability (Fig. 2C). This index has been shown to be the most representative measure of functional balance control in individuals with ankle instability [29]. The intraclass correlation coefficient (ICC) for these measures exceeds 0.81, confirming test-retest reliability [30].

Each test was repeated three times, and the average value was used for analysis. A trial was deemed invalid and repeated if the participant touched the safety handles, placed the contralateral foot on the platform, or lost balance entirely.

### Secondary outcomes

Isokinetic ankle muscle strength was evaluated using the Biodex System 4 Pro^®^ isokinetic dynamometer (Biodex Medical Systems Inc., USA), which offers high measurement reliability (ICC = 0.99-1.00) [31]. The system was calibrated before each use according to manufacturer specifications. Prior to testing, participants performed a 5-minute warm-up on a cycle ergometer and three submaximal practice repetitions at each test speed to ensure familiarization [32–34]. Muscle strength was assessed in concentric mode using the Biodex System 4 Pro^®^, which provides reliable and valid measurements of joint torque and is widely applied in studies of functional ankle instability and musculoskeletal rehabilitation [35, 36].

Muscle strength testing was performed with participants seated, the knee flexed at 45°, and the ankle positioned at neutral position (0°) (Fig. 1B). Gravity correction for limb weight was performed in accordance with the manufacturer’s guidelines before each test to ensure accurate torque measurements. To ensure accurate alignment, the mechanical axis of the Biodex dynamometer was carefully aligned with the anatomical axis of the ankle joint (approximately at the lateral malleolus) for each participant, following manufacturer instructions. The assessment included dorsiflexion/plantarflexion and eversion/inversion movement patterns, with concentric muscle contractions performed for all movement directions. The range of motion was set from − 10° to 30° for dorsiflexion/plantarflexion and from − 20° to 20° for eversion/inversion, based on common physiological ranges. For each pattern, participants completed two sets of five maximal repetitions. First at an angular velocity of 60°/s, followed by a 30-second rest, and then at 180°/s. Dorsiflexion and plantarflexion were tested first, followed by a 1-minute rest, after which eversion and inversion were assessed using the same velocity sequence. Participants were instructed to push and pull maximally throughout each repetition to capture true peak muscle performance. The following outcomes were recorded at both angular velocities: relative peak torque (RPT) (Nm/kg) for dorsiflexion and eversion; D/P torque ratio (%) and E/I torque ratio (%). RPT represents the peak torque normalized to body weight and serves as an indicator of muscle strength capacity. The D/P and E/I torque ratios reflect the balance between opposing muscle groups, which is critical for joint stabilization and injury prevention [13]. Higher values of the D/P torque ratio indicate a greater balance between the dorsiflexors and plantarflexors, whereas lower values suggest weakness or imbalances, potentially compromising ankle stability. Similarly, a higher E/I torque ratio indicates better strength balance between the evertors and invertors, which is crucial for dynamic stability, especially during activities that involve quick directional changes, while lower values could indicate an increased risk of ankle instability.

### Statistical analysis

All statistical analyses were performed using IBM SPSS Statistics, version 22.0 (IBM Corp., Armonk, NY, USA). The normality of the data distribution was assessed using the Shapiro-Wilk test before performing parametric tests. Categorical variables were expressed as frequencies and percentages, and differences between groups at baseline were evaluated using the chi-square test for categorical variables (e.g., sex, injured side) and independent two-sample t-tests for continuous variables (e.g., age, height, weight, BMI, CAIT score). Continuous variables were presented as mean ± standard deviation (SD). For inter-group comparisons at baseline and post-intervention, independent two-sample t-tests were used. To assess within-group changes from baseline to post-intervention, paired-sample *t*-tests were conducted. These parametric tests were selected due to their suitability for comparing group means when data are normally distributed. A two-tailed *P*-value < 0.05 was considered statistically significant for all analyses. Effect sizes (Cohen’s d) were used to assess the magnitude of between-group differences, with 0.2, 0.5, and 0.8 representing small, medium, and large effects, respectively. Corresponding 95% confidence intervals (CIs) indicate the precision of each estimate, where narrower CIs reflect greater consistency across participants.

## Results

### Participant recruitment and retention

**Fig. 3:**
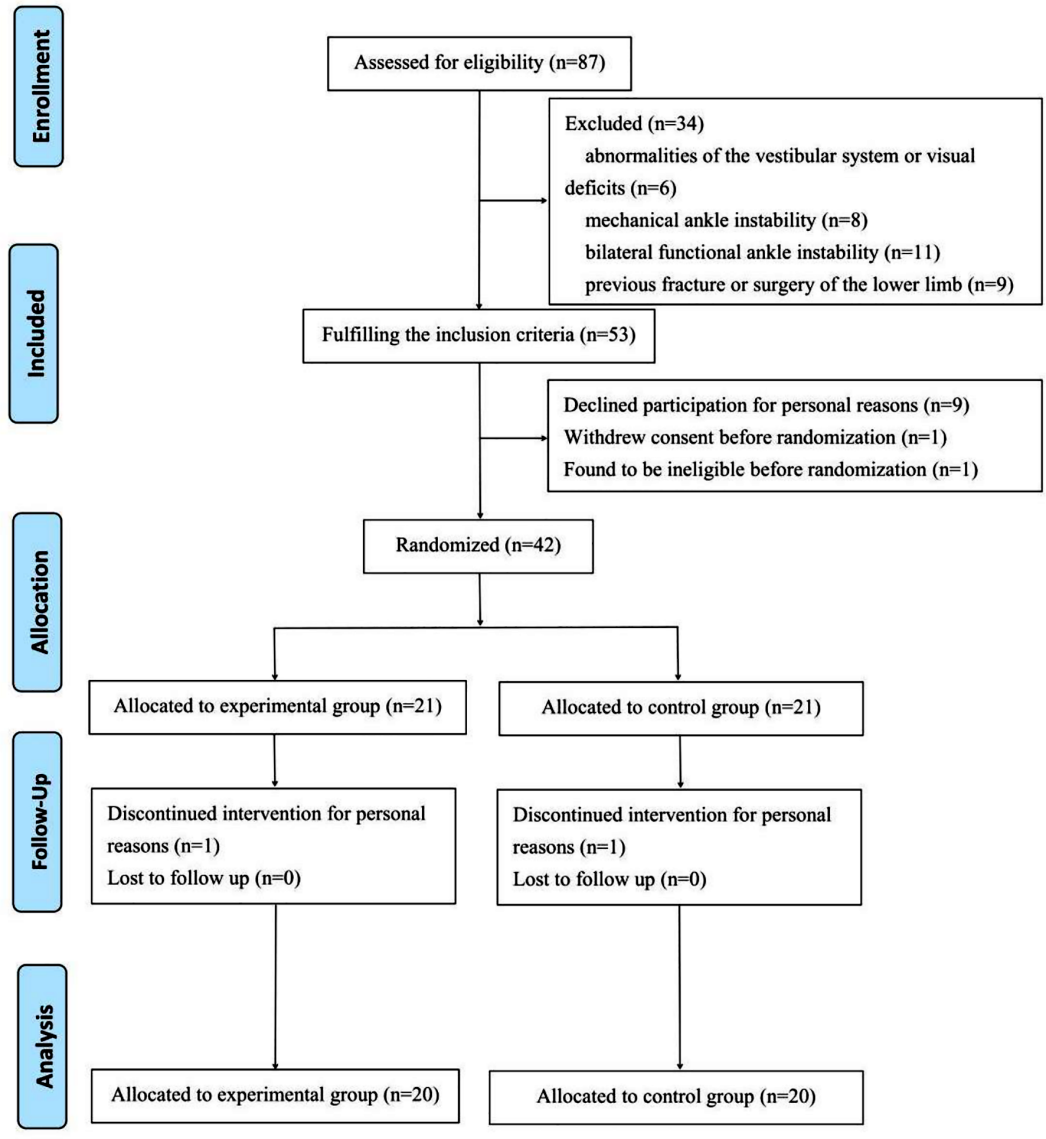
CONSORT flow diagram showing participant progression through the study. Of the 87 individuals assessed for eligibility, 34 were excluded due to: vestibular or visual abnormalities (*n* = 6), mechanical ankle instability (*n* = 8), bilateral functional ankle instability (*n* = 11), and prior lower limb fracture or surgery (*n* = 9). Of the 53 eligible participants, 11 were not randomized due to: personal refusal (*n* = 9), withdrawal of consent before randomization (*n* = 1), and later ineligibility (*n* = 1). A total of 42 participants were randomized equally into experimental (*n* = 21) and control (*n* = 21) groups. One participant in each group discontinued the intervention due to personal reasons. Final analysis included 20 participants in each group

A total of 42 unilateral FAI patients were enrolled, including 21 in the experimental group and 21 in the control group. One participant from the experimental group and one from the control group were lost to follow-up after 12-week intervention. Participants showed high adherence, with a mean training session attendance of 94.6% among study completers. A total of 20 participants in the experimental group and 20 participants in the control group were included in the final analysis. The number of participants retained in each community is shown in the CONSORT flow diagram (Fig. 3).

### Baseline characteristics of participants

There were no significant differences (*p* > 0.05) in baseline characteristics of FAI patients in the experimental group compared with the control group. Majority of participants were males (72.5%, *n* = 29). The average age was 26 years old (range from 20 to 35 years), the average height was 167 cm (range from 155 to 185 cm), and the average weight was 64 kg (range from 46 to 92 kg). The baseline characteristics are summarized in Table 2.

**Table 2:** Baseline demographic and clinical characteristics of the participants

Demographic characteristic	Experimental group (*n* = 20)	Control group(*n* = 20)	t/χ^2^	*P*
Age (years)	27.3 ± 4.8	25.3 ± 3.8	-1.470	0.150
Gender			0.125	0.723
Male	15	14		
Female	5	6		
Injured side			1.616	0.204
Left side	11	7		
Right side	9	13		
Height (cm)	168.5 ± 9.5	166.7 ± 7.5	0.682	0.499
Weight (kg)	63.2 ± 11.4	65.9 ± 16.0	-0.604	0.550
BMI (kg/m^2^)	22.5 ± 4.8	23.9 ± 6.2	-0.803	0.427
CAIT	20.1 ± 1.9	21.0 ± 1.9	-1.417	0.165

BMI, body mass index; CAIT, Cumberland Ankle Instability Tool; cm, centimeter; kg, kilogram; kg/m^2^, kilograms per square meter

### Comparison of postural control

At baseline, no significant differences were observed between the groups in total sway length (Cohen’s d = 0.27, 95% CI [-0.35, 0.89], *p* = 0.399) or total sway area (Cohen’s d = -0.44, 95% CI [-1.07, 0.19], *p* = 0.171). After the 12-week intervention, the experimental group showed a significant reduction in total sway area compared to the control group (Cohen’s d = 0.81, 95% CI [0.16, 1.45], *p* = 0.015), indicating improved static postural control. However, no significant group difference was detected in total sway length (Cohen’s d = 0.55, 95% CI [-0.08, 1.18], *p* = 0.090). Dynamic postural control, as measured by the total offset index, improved significantly in the experimental group compared to the control group (Cohen’s d = 2.06, 95% CI [1.29, 2.82], *p* < 0.001), suggesting a clinically meaningful enhancement in functional balance. Detailed information is shown in Table 3.

**Table 3:** Main outcome measures at baseline and 12 weeks in the experimental and control groups (Mean ± SD)

	Experimental group	Control group	Between-group difference
**Static postural control**			
**Primary outcome measure**			
**Total sway length (mm)**			
baseline	905.8 ± 46.6	923.2 ± 78.1	0.27 [-0.35, 0.89], *p* = 0.399
12 weeks	803.3 ± 43.0	824.6 ± 34.0	0.55 [-0.08, 1.18], ***p* = 0.090**
Within-group change (12 weeks)^*^	-1.23 [-1.80, -0.66],***p* < 0.001**	-1.56 [-2.20, -0.92], ***p* < 0.001**	
**Total sway area (mm** ^**2**^ **)**			
baseline	617.9 ± 35.5	602.6 ± 33.9	-0.44 [-1.07, 0.19], *p* = 0.171
12 weeks	511.0 ± 19.2	523.0 ± 8.4	0.81 [0.16, 1.45], ***p* = 0.015**
Within-group change (12 weeks)^*^	-2.62 [-3.05, -2.18], ***p* < 0.001**	-2.54 [-3.20, -1.89], ***p* < 0.001**	
**Dynamic postural control**			
**total offset index (°)**			
baseline	2.1 ± 0.1	2.2 ± 0.2	0.41 [-0.21, 1.04], *p* = 0.198
12 weeks	1.6 ± 0.1	1.8 ± 0.1	2.06 [1.29, 2.82], ***p* < 0.001**
Within-group change (12 weeks)^*^	-1.61 [-2.24, -0.98], ***p* < 0.001**	-2.72 [-3.42, -2.03], ***p* < 0.001**	
**Secondary outcome measure**			
**Ankle dorsiflexion RPT (60°/s) (Nm/kg)**			
baseline	29.84 ± 6.05	29.04 ± 6.38	-0.13 [-0.75, 0.49], *p* = 0.684
12 weeks	39.02 ± 6.04	33.87 ± 6.90	-0.79 [-1.44, -0.15], ***p* = 0.016**
Within-group change (12 weeks)^*^	3.86 [3.78, 3.93], ***p* < 0.001**	4.18 [4.02, 4.34], ***p* < 0.001**	
**Ankle dorsiflexion RPT (180°/s) (Nm/kg)**			
baseline	23.10 ± 5.92	21.79 ± 5.70	-0.23 [-0.85, 0.40], *p* = 0.479
12 weeks	31.65 ± 5.90	26.38 ± 6.30	-0.86 [-1.51, -0.22],***p* = 0.009**
Within-group change (12 weeks)^*^	3.71 [3.63, 3.79], ***p* < 0.001**	8.27 [8.20, 8.35], ***p* < 0.001**	
**Ankle eversion RPT (60°/s) (Nm/kg)**			
baseline	18.29 ± 4.12	28.03 ± 6.11	-0.10 [-0.72, 0.52], *p* = 0.750
12 weeks	17.89 ± 3.75	22.04 ± 4.69	-1.10 [-1.76, -0.43], ***p* = 0.001**
Within-group change (12 weeks)^*^	2.16 [1.98, 2.33], ***p* < 0.001**	3.00 [2.78, 3.22], ***p* < 0.001**	
**Ankle eversion RPT (180°/s) (Nm/kg)**			
baseline	11.55 ± 2.72	12.00 ± 2.91	0.16 [-0.46, 0.78], *p* = 0.620
12 weeks	20.29 ± 3.79	16.28 ± 3.32	-1.13 [-1.79, -0.46], ***p=*0.001**
Within-group change (12 weeks)^*^	1.77 [1.44, 2.11], ***p* < 0.001**	4.38 [4.15, 4.61], ***p* < 0.001**	
**D/P torque ratio (60°/s) (%)**			
baseline	28.7 ± 2.7	29.3 ± 1.6	0.27 [-0.35, 0.90], *p* = 0.392
12 weeks	34.6 ± 2.6	32.5 ± 2.4	-0.84 [-1.49, -0.20], ***p* = 0.011**
Within-group change (12 weeks)^*^	1.01 [0.33, 1.70], ***p* < 0.001**	1.86 [1.34, 2.38], ***p* < 0.001**	
**D/P torque ratio (180°/s) (%)**			
baseline	26.1 ± 4.1	26.9 ± 2.1	0.24 [-0.38, 0.86], *p* = 0.447
12 weeks	32.7 ± 1.3	29.8 ± 1.3	-2.18 [-2.96, -1.39], ***p* < 0.001**
Within-group change (12 weeks)^*^	1.25 [0.66, 1.84], *p* < 0.001	1.62 [1.05, 2.18], *p* < 0.001	
**E/I torque ratio (60°/s) (%)**			
baseline	79.7 ± 1.5	80.1 ± 1.8	0.25 [-0.37, 0.87], *p* = 0.439
12 weeks	88.3 ± 1.0	83.9 ± 0.9	-4.50 [-5.67, -3.34], ***p* < 0.001**
Within-group change (12 weeks)^*^	1.79 [1.11, 2.48], ***p* < 0.001**	4.93 [4.34, 5.53], ***p* < 0.001**	
**E/I torque ratio (180°/s) (%)**			
baseline	70.1 ± 1.8	71.7 ± 3.2	0.24 [-0.38, 0.86], *p* = 0.455
12 weeks	79.7 ± 1.6	74.5 ± 1.0	-3.88 [-4.93, -2.83], ***p* < 0.001**
Within-group change (12 weeks)^*^	0.91 [0.42, 1.41], ***p* = 0.001**	3.02 [2.28, 3.75], ***p* < 0.001**	

Cohen’s d values and their 95% confidence intervals are reported alongside *p*-values to indicate effect sizes and precision. mm, millimeter; mm^2^, square millimeters; RPT, relative peak torque; D/P, dorsiflexion/plantarflexion; E/I, eversion/inversion; s, second; Nm/kg, newton meter per kilogram

^*^ Values represent effect sizes (Cohen’s d) for within-group pre-post changes. Bolded p-values indicate statistical significance (*p* < 0.05)

### Comparison of isometric muscle strength test

At baseline, there were no statistically significant differences between the two groups in ankle dorsiflexion and eversion RPT, as well as in the D/P and E/I torque ratios (all *p* > 0.05). Following the 12-week intervention, the experimental group demonstrated significantly greater improvements in all ankle strength parameters compared to the control group. Specifically: Dorsiflexion RPT at 60°/s showed a moderate between-group effect size (Cohen’s d = − 0.79, 95% CI [− 1.44, − 0.15], *p* = 0.016); Eversion RPT at 180°/s exhibited a large effect size (Cohen’s d = − 1.13, 95% CI [− 1.79, − 0.46], *p* = 0.001); D/P torque ratio at 180°/s presented a very large effect size (Cohen’s d = − 2.18, 95% CI [− 2.96, − 1.39], *p* < 0.001); E/I torque ratio at 60°/s yielded an extremely large effect size (Cohen’s d = − 4.50, 95% CI [− 5.67, − 3.34], *p* < 0.001). These findings not only reflect statistically significant differences, but also indicate substantial and clinically meaningful improvements in ankle strength balance. Detailed information is shown in Table 3.

## Discussion

This randomized controlled trial investigated the effects of a 12-week isokinetic eccentric training program on postural control and ankle strength in individuals with FAI. Our findings confirmed the hypothesis that eccentric training would lead to greater improvements in neuromuscular strength and functional balance compared to concentric training.

Notably, dynamic postural control, indicated by the total offset index, improved significantly in the eccentric group, reflecting enhanced balance during movement and responses to perturbations, which are common deficits in individuals with FAI. Substantial gains in dorsiflexion and eversion peak torque were observed, especially at higher angular velocities where neuromuscular demands are elevated. These muscle groups play a critical role in ankle stability and proprioceptive control during functional tasks [37]. Improvements in D/P and E/I torque ratios, markers of strength symmetry between opposing muscle groups, indicate better neuromuscular coordination. These findings align with previous literature suggesting that eccentric training elicits more robust neuromuscular adaptations than concentric-only regimens, particularly in populations with chronic joint instability [38, 39].

At 180°/s, the D/P torque ratio exhibited a very large effect size (Cohen’s d = -2.18), while the E/I torque ratio at 60°/s demonstrated an exceptionally large effect size (Cohen’s d = -4.50), indicating that isokinetic eccentric training had a substantial impact on both strength development and postural control compared to concentric training. Specifically, dorsiflexion RPT in the eccentric training group significantly increased from 29.84 to 39.02 Nm/kg at 60°/s and from 23.10 to 31.65 Nm/kg at 180°/s. In contrast, the control group showed only modest improvements. Regarding eversion strength, the most pronounced gain in the eccentric group was observed at 180°/s, with RPT increasing from 11.55 to 20.29 Nm/kg, compared to an increase from 12.00 to 16.28 Nm/kg in the control group. These results suggest that isokinetic eccentric training is particularly effective in enhancing dorsiflexor strength across both low and high velocities and significantly improves evertor strength under high-speed conditions, an adaptation crucial for improving dynamic ankle stability in individuals with functional ankle instability.

Eccentric contractions provide greater mechanical loading and prolonged muscle-tendon activation, which stimulate both peripheral and central adaptations. These adaptations include improved motor unit recruitment, heightened muscle spindle sensitivity, and increased cortical excitability [40–42]. Such neural and muscular changes likely account for the superior postural outcomes observed in the eccentric training group. In terms of balance assessment, differences between sway area and sway length may reflect distinct biomechanical functions. Total sway area quantifies the spatial dispersion of the center of pressure (COP), serving as an indicator of the overall “envelope” of postural stability. A significant reduction in sway area, as observed in the eccentric group, suggests enhanced spatial control and a decrease in gross sway, reflecting improved global balance capacity. In contrast, total sway length measures the cumulative distance traveled by the COP, which captures more subtle postural adjustments over time. The lack of significant between-group differences in sway length may indicate that the eccentric group exhibited more frequent and precise corrective movements, rather than a true instability. Previous studies have shown that sway area is more responsive to balance interventions, whereas sway length may increase due to enhanced neuromuscular reactivity [28].

Given the sensorimotor deficits characteristic of FAI, eccentric training offers specific advantages by challenging joint stability through controlled lengthening contractions [43]. This could result in greater improvements in proprioceptive acuity and reflexive stabilization [44]. Indeed, the observed effect sizes in strength ratios (e.g., D/P torque ratio at 180°/s, d = -2.18; E/I torque ratio at 60°/s, d = -4.50) far exceed conventional thresholds for clinical significance, suggesting not only meaningful strength improvements but also potential reductions in injury recurrence risk [45, 46]. Functionally, these neuromuscular gains may support better athletic performance and everyday activities requiring rapid directional changes [47]. The enhancement in muscle balance across agonist-antagonist groups likely improves joint congruency and reduces compensatory loading on passive structures, such as ligaments [48]. Additionally, better strength symmetry may contribute to more efficient energy transfer during gait and jumping tasks, which are frequently impaired in individuals with FAI [49].

Despite promising results, this study has limitations. First, the sample size (*n* = 40), while sufficiently powered for medium-to-large effects, may limit generalizability. Second, the 12-week intervention captured short-term improvements but did not assess the sustainability of gains. Third, the absence of long-term follow-up and return-to-activity measures limits the evaluation of functional recovery. In addition, assessments were performed in a controlled laboratory setting, which may not fully represent real-world performance. Although improvements in torque and balance were observed, neuromuscular activation was not directly measured, leaving mechanistic explanations inferential. Future research should replicate these findings in larger, more diverse populations, including athletes and older adults. Longitudinal studies are warranted to assess the durability of effects and their impact on injury prevention and performance. The use of neuromechanical assessments (e.g., EMG, motion capture) could further clarify the effects of eccentric training on motor control. Comparative studies with other modalities, such as perturbation or balance training, would also help define optimal rehabilitation strategies. Finally, due to the distinct nature of the intervention protocols, blinding of participants and therapists was not feasible, which may introduce performance bias.

Statistical considerations must also be noted. Although we did not apply formal corrections for multiple testing (e.g., Bonferroni), we interpreted findings cautiously. Sample size estimation was based solely on dorsiflexor torque, so secondary outcomes such as eversion strength and torque ratios, while statistically significant and showing large effect sizes, should be interpreted with caution. These findings warrant confirmation in future studies with larger sample sizes to ensure reproducibility and generalizability. Although the experimental group showed reduced sway length compared to controls, the difference was not statistically significant (d = 0.55, *p* = 0.090). Despite the moderate effect size, the clinical relevance is uncertain, and the finding should be interpreted with caution. The change may reflect minor postural adjustments rather than meaningful functional improvement. While some effect sizes were exceptionally large (e.g., D/P torque ratio at 180°/s, Cohen’s d = -2.18; E/I torque ratio at 60°/s, Cohen’s d = -4.50), such magnitudes are uncommon in clinical research and may be influenced by outliers, individual variability, or the relatively small sample size. These values should therefore be interpreted with caution and confirmed in future studies with larger and more diverse populations.

To aid clinical translation, Table 4 summarizes the practical implementation of the isokinetic eccentric training protocol used in this study. The structured progression (12 weeks, 70–90% MVIC, progressive isokinetic velocity ramping) offers a replicable, evidence-based approach for enhancing ankle strength, postural control, and torque symmetry in patients with unilateral FAI. This protocol can be readily integrated into outpatient rehabilitation settings equipped with isokinetic dynamometers.

**Table 4:** Clinical implementation summary for isokinetic eccentric training in FAI

Parameter	Recommendation
Target population	Adults with unilateral FAI
Training duration	12 weeks
Frequency	3 sessions/week
Intensity progression	Weeks 1–4: 70% MVIC; Weeks 5–8: 80% MVIC; Weeks 9–12: 90% MVIC
Angular velocities	60°/s → 120°/s → 180°/s → 120°/s → 60°/s
Movements	Dorsiflexion/Plantarflexion + Eversion/Inversion
Contraction type	Isokinetic eccentric only (unilateral training)
Repetitions per session	2 sets of 50 reps (10 reps per velocity step)
Rest intervals	1 min between speeds; 3 min between sets
Equipment required	Biodex System 4 Pro^®^ or equivalent isokinetic dynamometer

FAI, functional ankle instability; MVIC, maximal voluntary isometric contraction

In conclusion, this study demonstrates that structured isokinetic eccentric training yields significant improvements in ankle strength, torque symmetry, and postural control in individuals with FAI. These findings support incorporating eccentric training into rehabilitation protocols aimed at restoring neuromuscular function and preventing recurrence. Given the substantial observed effect sizes and strong theoretical basis, this approach is particularly suited for outpatient settings with isokinetic equipment. Future studies should investigate the long-term effects of this intervention on reinjury risk, return-to-sport timelines, and functional durability.

## Data Availability

No datasets were generated or analysed during the current study.
